# Structural Perspective of Gliadin Peptides Active in Celiac Disease

**DOI:** 10.3390/ijms21239301

**Published:** 2020-12-06

**Authors:** Lucia Falcigno, Luisa Calvanese, Mariangela Conte, Merlin Nanayakkara, Maria Vittoria Barone, Gabriella D’Auria

**Affiliations:** 1 Department of Pharmacy, University of Naples Federico II, Via Mezzocannone 16, 80134 Naples, Italy; luisa.calvanese@unina.it; 2 Department of Translational Medical Science, Section of Pediatrics, University of Naples Federico II, 80131 Naples, Italy; maryconte_92@hotmail.it (M.C.); merlinnanayakkara@yahoo.it (M.N.); mv.barone@unina.it (M.V.B.); 3 European Laboratory for the Investigation of Food Induced Diseases (ELFID), University of Naples Federico II, 80131 Naples, Italy

**Keywords:** P31–43, NMR structure, HLA-DQ, innate and adaptive immune systems

## Abstract

Gluten fragments released in gut of celiac individuals activate the innate or adaptive immune systems. The molecular mechanisms associated with the adaptive response involve a series of immunodominant gluten peptides which are mainly recognized by human leucocyte antigen (HLA)-DQ2.5 and HLA-DQ8. Other peptides, such as A-gliadin P31–43, are not recognized by HLA and trigger innate responses by several routes not yet well detailed. Among the gluten fragments known to be active in Celiac disease, here we focus on the properties of all gluten peptides with known tri-dimensional structure either those locked into HLA-DQ complexes whose crystals were X-ray analyzed or characterized in solution as free forms. The aim of this work was to find the structural reasons why some gluten peptides prompt the adaptive immune systems while others do not, by apparently involving just the innate immune routes. We propose that P31–43 is a non-adaptive prompter because it is not a good ligand for HLA-DQ. Even sharing a similar ability to adopt polyproline II structure with the adaptive ones, the way in which the proline residues are located along the sequence disfavors a productive P31–43-HLA-DQ binding.

## 1. Introduction

In celiac individuals, the peptides deriving from the digestion of gluten proteins of wheat, barley, oats, and rye cause immune reactions and thus inflammation of the intestinal mucosa. The enzymatic hydrolysis of the hundred proteins contained in the gluten causes the release of peptides in the gut, some of which are able to activate the innate or adaptive immune systems. The aim of this work is to identify the structural characteristics that make different gliadin peptides competent for one or the other of the immune systems.

Up until today, the molecular mechanism associated to the innate immune response to gluten peptides is less detailed than that associated to the adaptive response. Previous research about the chain of pathological events related to Celiac disease (CeD) led to the identification of human leucocyte antigen (HLA) as the molecular player deputed to present the antigenic peptides to T-cells [1]. The interesting history of how HLA was discovered, and who discovered it, was passionately reviewed by E. Thorsby [2].

In CeD, the αb T-cells antigen receptor (TCR) specifically recognizes de-amidated gluten peptide when presented by HLA-DQ2.5 (expressed by 90% of CeD patients), HLA-DQ8 or HLA-DQ2.2 [3]. Interestingly, the adaptive response is directed versus just few epitopes among the plethora of very similar gluten peptides produced by digestion and resident in the gut [4]. These immunodominant epitopes are HLA-DQ2.5-glia-α1a, DQ2.5-glia-α2, DQ2.5-glia-ω1, DQ2.5-glia-ω2 [5,6] and DQ8-glia-α1 [5,7]. Indeed, other gliadin and glutenin peptides are also involved in CeD immunoreactions. A coherent selection of DQ2.5, DQ2.2, DQ8 and DQ8.5 restricted epitopes recognized by CD4+ T cells and a new nomenclature for this selection was published in 2012 and updated in 2020 by Ludvig M. Sollid [4,8]. The first crystallographic structures of gluten peptide-HLA-DQ and peptide-HLA-DQ/T-cell-receptor, binary and ternary complexes, respectively, were published starting from 2004 (Protein Data Bank (PDB) code 1S9V, [9]) and 2012 (PDB code 4GG6, [7]), respectively. Since then, the X-ray images allowed the structural reasons why HLAs recognize specific peptides and TCRs recognize specific HLA-peptide complex in CeD to be explained. Beside the immunodominant peptides (DQ2.5-glia-α1a, DQ2.5-glia-α2, DQ2.5-glia-ω1, DQ2.5-glia-ω2 and DQ8-glia-α1), a number of gluten fragments are known to provoke inflammation of the intestinal mucosa by stress/innate but not adaptive immune response. Why they are dangerous and how they work in the gut of CeD patients is still not fully deciphered. One of the most investigated peptides belonging to this category is A-gliadin P31–43, L^31^GQQQPFPPQQPY^43^ [10]. This peptide, not presented by either HLA-DQ2 or HLA-DQ8 [11], activates innate immune response following several routes. A series of studies show that this gliadin peptide is able to activate interferon-α (IFN-α), a mediator of the immune response in the intestine of CeD patients, and an enterocyte cell line, CaCo-2 [12]. In co-operation with a viral ligand, P31–43 is able to interfere with endocytic trafficking thus activating the toll like receptor 7 (TLR7) pathway [12]. This prompted the idea that, together with viral infections, alimentary proteins able to mimic and potentiate the innate immune response to viruses, can trigger an autoimmune disease such as CeD.

Other studies show how P31–43, by binding NBD1 domain of cystic fibrosis trans-membrane conductance regulator (CFTR), an anion channel involved in the epithelial adaptation to environmental stress, impairs CFTR function that in turn generates epithelial stress, tissue transglutaminase and inflammasome activation [13]. Moreover, recent studies propose that the toxic behavior of P31–43 in CeD individuals is due to its ability to self-aggregate and form oligomers able to induce NLRP3 Inflammasome/Caspase 1- dependent mucosal damage in the small intestine [14]. Finally, a very recent study proposes a synergy among tissue transglutaminase TG2, anti-TG2 antibodies and peptide P31–43 in CeD starting and progression [15]. A complete review about the multiple pro-inflammatory effects induced by the gliadin peptide P31–43 is currently in press [16].

Recently, simulated docking experiments suggest that the reason why P31–43 does not work into the adaptive immune circuit is that P31–43 is not a good binder for HLA-DQ, in particular for DQ2 [17]. In the present contribution, we report our analysis of all the known X-ray binary and ternary complexes involving gluten fragments in HLA-DQ and HLA/TCR complexes with the aim to catch, if any, the structural properties that make specific gluten fragments immune-dominant epitopes and some others, like gliadin P31–43, non-ligands.

## 2. Results

### 2.1. More Details about the P31–43 Structure Free in Solution

We recently published the structural properties of P31–43 obtained by proton nuclear magnetic resonance (NMR) in solution [17]. This peptide, being very rich in prolines, shows specific conformational properties. Indeed, in peptides and proteins, each peptide bond connecting pairs of adjacent residues generally adopts *trans* configuration (dihedral angle ω of the backbone Cαi − C’i – Ni + 1 – Cαi + 1 atoms is ∼180°) because, due to the smaller steric hindrance between the side chains of the adjacent amino acids, it is on average more stable of about 2.5 kcal mol^−1^ of the *cis* form (ω ∼ 0 °) [18,19]. The *trans* isomer is also kinetically stabilized in reason of an isomerization barrier *trans* → *cis* of about 20 kcal mol^−1^ [18]. However, in the Xxx-Pro bond (Xxx = any residue), frequently found in gluten peptides, the *trans* and *cis* conformations which only differ by ∼0.5 kcal mol^−1^ are practically iso-energetic, and show inter-conversion barrier as low as 13 kcal mol^−1^ [19]. This means that while in proteins the three-dimensional structure forces the Xxx-Pro bond mainly in *trans* configuration (*cis* configuration is observed in just ∼5 % of cases [19,20]), in peptides the Xxx-Pro bond visits both the configurations. Thus, while in peptides with no Pro residues all peptide bonds are in *trans* configuration (in the following ‘all *trans*’), Pro bearing peptides may adopt *trans* and *cis* configuration at each Xxx-Pro bond in the sequence. Other relevant issue for peptides that, as gluten fragments, contain Pro, is that the cyclic structure of proline’s side chain locks the proline φ angle at approximately −65° [21], a value that favors polyproline II structure (PPII: φ ∼ −75°, ψ ∼ 150°).

How peptide P31–43 works in CeD depends on its structure and on the structure of its molecular targets. P31–43 contains 13 residues including 4 prolines. This means that it can potentially adopt 4^2^ different structures due to the different *cis*-*trans* combinations of the four Xxx-Pro bonds.

**Figure 1:**
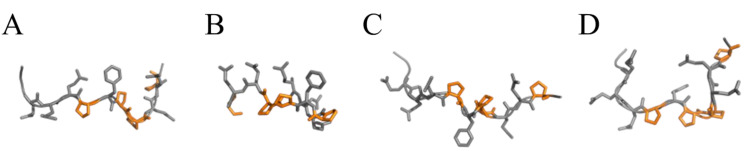
P31–43 (L^31^GQQQPFPPQQPY^43^) modelled in (**A**) ‘all *trans*’, (**B**) *cis* Gln35-Pro36, (**C**) *cis* Phe37- Pro38, and (**D**) *cis* Pro38-Pro39 configurations. Proline residues are colored in gold.

The structural differences between the numerous forms are relevant, as can be appreciated in Figure 1, where P31–43 modelled with all the four prolines ‘in *trans*’ or Pro36, Pro38, and Pro39 singularly in *cis* configuration, is shown. Still, other structures may come from *cis* Pro42 and all possible combinations of two, three or all four *cis* bonds. 

P31–43 conformational behavior in solution has been investigated in recent times by proton nuclear magnetic resonance [17], circular dichroism, fluorescence spectroscopy, several techniques characterizing self-aggregate forms of the peptide and molecular dynamics studies in oligomeric and monomeric forms of the peptide [14,22,23].

**Figure 2:**
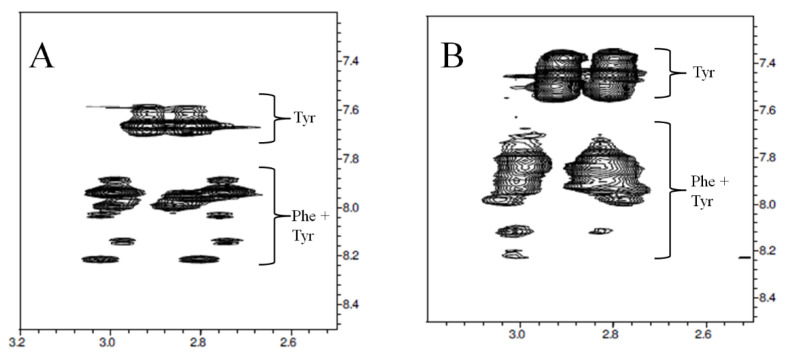
(**A**) Expansion of the Tocsy map of P31–43 in water showing the NH-ββ’CH2 scalar correlation peaks of Phe36 and Tyr43 residues. (**B**) Same map expansion of the Tocsy experiment performed for P31–43 in SDS 150 mM.

The conformational analysis of P31–43 in aqueous solution performed through NMR [17] shows that, although the sequence contains a single Phe and a single Tyr residue, each of these residues exhibits well more than two spin systems, indicating that Phe and Tyr are not only affected by the different *cis*/*trans* configurations of the peptide bond in which they are involved but also by the different configuration of the other Xxx-Pro bonds further away in the sequence. This phenomenon can be observed in the expansion of the reported Tocsy scalar correlation spectrum of P31–43 reported in Figure 2.

**Figure 3:**
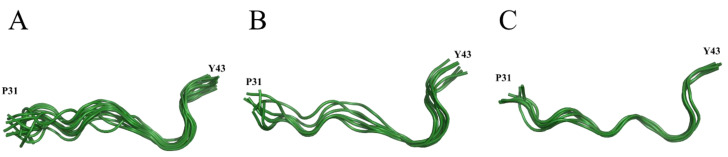
First three clusters of P31–43 NMR structures (ribbon representation) as clustered by Chimera software [24]. (**A**) Cluster 0, the most populated cluster collecting 14 structures over forty; (**B**) cluster 1 with 7 structures over forty; (**C**) cluster 2 with 7 structures over forty.

The complexity of the two-dimensional NMR proton spectra confirmed that the peptide adopts a variety of *cis*/*trans* combinations of Xxx-Pro bonds, potentially all 4^2^ theoretically predictable structures with different percentages and in slow inter-conversion kinetic at the NMR time scale. The analysis allows the identification of the ‘all *trans*’ structure and to describe its conformational propensities. Starting from measurements of NOE dipolar coupling effects, NMR analysis allows the time averaged distances between pairs of hydrogen nuclei placed along the sequence to be estimated. Once the inter-protons distances (NMR distances) are determined, they are used as experimental constraints for calculating a number of peptide structures compatible with them. The greater the number of the experimental constraints, the more accurately the secondary structure of the peptide is defined. The structures that best adhere to the NMR distances, taken all together, represent the conformational behavior of the peptide in solution. A way to catch how wide the distribution of conformations is, is to group the structures by similarity. The higher the number of clusters, the wider the structures distribution is and the higher the peptide flexibility is. Moreover, the higher the population of a single cluster, the higher the weight of that structure is in describing the conformational preferences of the peptide. The forty P31–43 structures obtained by NMR analysis (deposited with PDB code 6QAX), once clustered by resemblance, revealed six different groups of which the first three most populated ones, counting for more than 50% of the entire structure population, are shown in Figure 3.

It is interesting to note that none of the NMR structures showed PPII torsion angles along the entire sequence. Nevertheless, the propensity to adopt this motif is clear in each cluster, particularly in cluster 0 (Figure 3A), where segments of 2–3 consecutive amino acids, differently localized in different structures, adopt PPII conformation. This result is in accordance with circular dichroism measurements showing that the peptide tends to adopt a PPII structure in equilibrium with random structures [14,23]. The peptides that enter or are generated in cells or tissues interact with membranes, receptors, and other molecules, triggering processes with a favorable or unfavorable outcome for the host organism. To characterize the ability of P31–43 to interact with biological membranes, the peptide was incubated with SDS, a membrane mimic, and the interaction tested by fluorescence analysis [25]. This same interaction was also tested by using the NMR technique [17]. Although SDS micelles do not represent the best system for mimicking human cell membranes, they have been used to verify whether the interaction with a micellar surface had the ability to restrict the variety of conformations exhibited by P31–43 in free form. The spectrum shown in Figure 2B shows wider peaks with respect to those visible in the analogous spectrum of Figure 2A. The widening of the peaks confirms the interaction between peptide and SDS, since it indicates that the peptide tumbles in solution at lower frequencies than those of the free peptide, that is it rolls at the tumbling frequencies of the micelles to which it adheres. Nevertheless, the fact that the number of signals is not significantly reduced (compare Figure 2A,B) indicates that the micelles formed by SDS are not able to stabilize particular conformations of P31–43 and that ultimately the peptide-SDS interaction is non-specific.

### 2.2. Structure of Gluten Peptides Able to Bind or Not in the HLA-DQ Groove

The physiological manifestations linked to CeD are partly due to the activation by some gliadin peptides of the gluten-reactive CD4+ T-cells of the intestinal mucosa. The molecular mechanism is known and foresees that (i) the gliadin peptide is recognized and deamidated to one of its Gln by TG2 [26]; (ii) the deamidation transforms the gluten peptide in a better binder for HLA-DQ [27]; (iii) the deamidated peptide is recognized mainly by the type II HLA-DQ2 or DQ8 expressed by celiac subjects [28,29]; (iv) the complex is recognized by the CD4+ T cell with consequent induction of proliferative effects.

**Figure 4:**
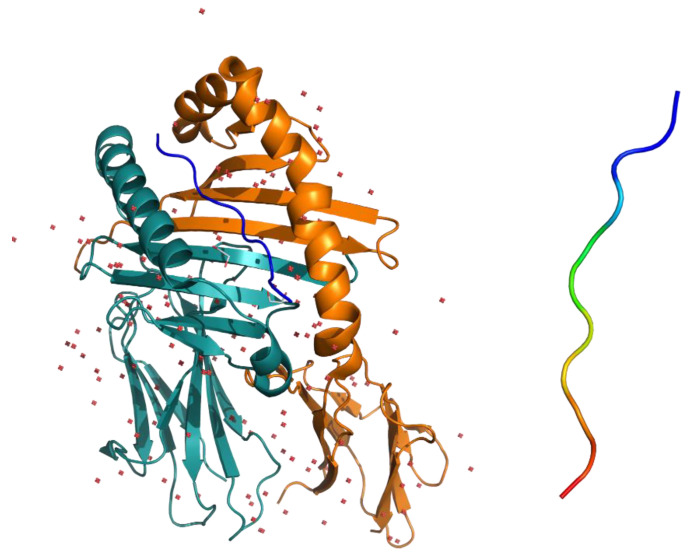
**(Left**) X-ray structure of HLA-DQ2.5-glia-α1 complex (PDB code 1S9V); (**Right**) glia-α1 structure as found in the complex (color code: blue = N-terminal end, red = C-terminal end.).

The first crystallographic structure of the complex formed by the soluble portion of HLA-DQ2 and the gliadin peptide α1 (DQ2-glia-α1 = PFPQPE^6^LPY, nomenclature of gliadin epitope following Sollid et al. [8]) was published by Ludvig M. Sollid and co-authors in 2004 [9]. HLA-DQ2 general folding is typical of the MHC class II family which HLA belongs to. The groove that houses the peptide α1 has a typical architecture formed by a β-sheet platform on which two parallel α-helices sit on as walls (Figure 4), while the peptide glia-α1 adopts a conformation close to PPII (all *trans* peptide bonds, φ ≈ −75°, ψ ≈ 150°). It must be noted that having four proline residues, the α1 peptide lacks as many as 4 NH amides, possibly useful for establishing hydrogen bonds with the groove. Nevertheless, as underlined by Chu-Young Kim and co-authors [9], α1 peptide docks into the binding pocket in a way (i.e., register) that is the best one for maximizing the network of H-bonds (particularly relevant to those involving Glu6 side chain), that together with charge and hydrophobic interactions make the complex stable (see Figure 3 of [9]). 

Different HLA-DQs show different specificity for different gluten peptides because of the diversity of amino acid side-chains anchoring the peptide into the groove.

The list of PDB structures of binary and ternary complexes involving HLA-DQ, gluten peptides and T-cell receptor updated to October 2020 is reported in Table 1.

**Figure 5:**
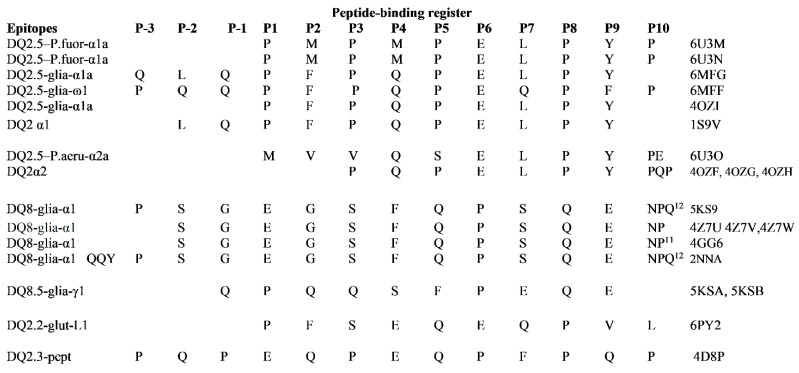
Peptide binding register of immunodominant peptides in complexes with HLA-DQ and T-cell receptor.

Peptide binding register of immunodominant peptides in these complexes with HLA-DQ and T cell receptor is shown in Figure 5.

**Figure 6:**
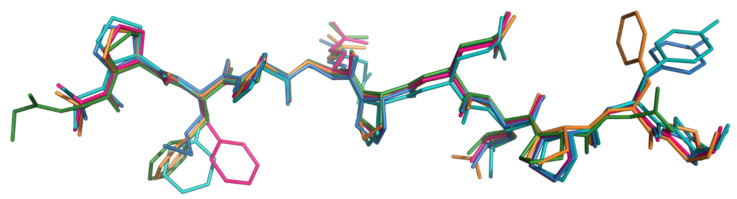
Superimposition of DQ2α1 (from binary complex, PDB code 1S9V, forest), DQ2.5-glia-α1a (from binary complex, PDB code 6MFG, hot pink) DQ2.5-glia-α1a (from ternary complex, PDB code 4OZI, teal), DQ2.5-glia-ω1 (from ternary complex, PDB code 6MMF, orange), DQ2.5–*P.fluor*-α1a (from binary complex, PDB code 6U3M, blue).

We isolated the peptide ligands from the complexes and compared them with the aim to find, if any, the structural properties that make specific gluten fragments immunodominant epitopes and some others, like gliadin P31–43, non-ligands. The superimposition of four DQ2.5-glia-α1 X-ray structures is shown in Figure 6. Even though belonging to different data collections, the α1 conformation in the HLA-DQ2.5 groove is strictly reproduced. Some structural differences are just found in the orientation of Phe and Tyr side chains in P2 and P9 sites of the DQ2.5 binding pocket, respectively. In the bundle in Figure 5, we also included DQ2.5-glia-ω1 (colored in orange). This gliadin epitope differs from α1 for having Leu instead of Gln at P7 and Phe instead of Tyr at P9 (further than two other less relevant differences at P-3 and P-1 sites, see Figure 5). A visual comparison shows that four diverse residues do not change the glia-ω1 backbone conformation respect to that of glia-α1. Very recently, Petersen et al. [30] published the X-ray structures of HLA-DQ2.5 complex to peptide fragments from bacteria proteins with high sequence homology with glia-α1 and α2. The gliadin mimic from *Pseudomonas fluorescence*, DQ2.5–*P.fluor*-α1a, differs from glia-α1a for having two Met residues instead of Phe and Gln in P2 and P4 sites. As it can be seen in Figure 6, the mimic peptide, represented as blue sticks, adopts the backbone structure similar to those adopted by glia-α1 ligands and glia-ω1. The comparison in Figure 6 demonstrates that the topology of the binding groove shapes all ligands in a PPII-like conformation. The quantitative measures of the similarity between superimposed atomic coordinates of the peptides, i.e., the root mean square deviation (RMSD) values, are listed in Table S1 of the Supplementary Materials.

**Figure 7:**
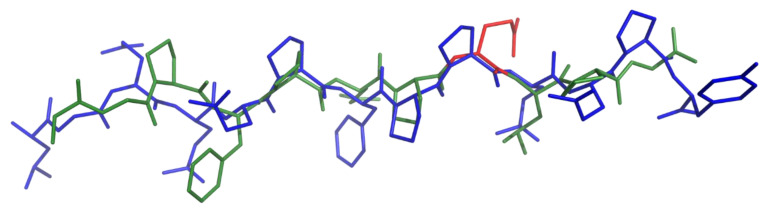
Superimposition of DQ2-α1 (from binary complex PDB code 1S9V, colored in forest with its glutamic acid at P6 colored in red) with P31–43 modelled in PPII structure (blue).

P31–43 is known not to be a ligand either for HLA-DQ2.5 or DQ8 [11]. Moreover, the in silico experiments performed by docking P31–43 onto HLA-DQ2.5 show that the peptide in the experimental structure, as well as modelled in PPII, engages in both conditions a number of interactions with the binding groove lower than that established by glia-α1 [17]. A representation of such finding is visible in Figure 7 where DQ2.5 glia-α1 and P31–43 in PPII structure are superimposed. In this case, the accordance between the two peptides shows an RMSD value of 1.30 Å, well above the RMSD values calculated between DQ2.5-glia-α1a and DQ2.5–*P.fluor*-α1a or between Q2.5-glia-α1a and DQ2.5-glia-ω1 (Table S1). The PPII arrangement of P31–43 is not enough for a correct positioning of Pro residues into the groove because, even if P31–43 bears the same number of Pro as in DQ2.5-glia-α1, those are differently localized along the sequence. The consequence is that P31–43, differently from glia-α1, does not find any useful register to establish the network of H-bonds needed to stabilize the complex.

The gluten peptides that bind to HLA-DQ8 are different from those recognized by HLA-DQ2.5. Particularly, DQ8 epitopes contain no more than two prolines in the binding core in respect to the three-four Pro residues of HLA-DQ2.5 core epitopes and, importantly for the binding, Glu residues at P1 and P9 sites. 

**Figure 8:**
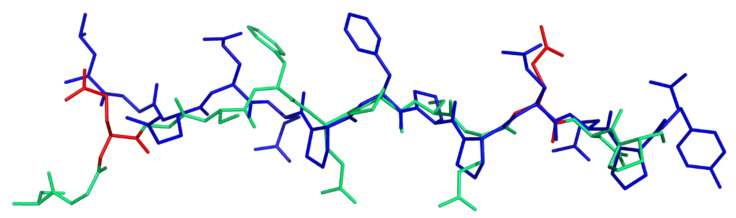
Superposition of DQ8-glia- α1 (from binary complex PDB code 2NNA, colored in green with Glu residues at P1 and P9 colored in red) with P31–43 modelled in PPII structure (blue).

The superimposition of DQ8-glia-α1 peptides, as extracted from thebinary and ternary X-ray complexes, shows a strict reproducibility among the ligand structures. HLA-DQ8 binding groove shapes the ligand in a PPII-like conformation. Again, to catch the differences in binding ability between DQ8-glia-α1 and P31–43, we consider the superimposition of the first with the second modelled in PPII (RMSD 1.1 Å). As it can be observed in Figure 8, P31–43 (blue sticks) shows residue side-chains totally different both for nature and localization from those of DQ8-glia-α1 (green), thus failing in stabilizing the right interactions with the groove.

## 3. Discussion

Here we report the analysis of all structurally characterized gluten fragments active in CeD with the objective to find the structural reasons that make specific gluten fragments players in the immune-adaptive system by binding HLA-DQs molecules, and some others, like gliadin P31–43, non-ligands of HLA, and instead players in innate immune routes.

The immunodominant gliadin peptides productively interact and bind type II HLA receptors because the process is energetically favored. Differently, P31–43, very similar by composition to the immunodominant gliadin peptides, does not bind type II HLAs, meaning that for some reasons the binding process is disfavored. In order to understand why, we considered comparing the structural properties of the peptides belonging to both categories to be useful, that is the binders and not-binders of HLA-DQ. The NMR study of P31–43 in solution shows that the peptide exhibits *cis*/*trans* isomerism at Xxx-Pro sites and, for each isomer, the peptide adopts a distribution of different conformers. The ‘all *trans*’ structures represent at least 60–65% of the entire population and exhibit a clear tendency to adopt PPII conformation [17], i.e., that ‘required’ by HLA binding. Dynamic light scattering (DLS) measurements showed that P31–43 forms aggregates in solution [14]. For that, by considering NMR and DLS results together, the emerging picture is that P31–43 lives in solution distributed between monomeric and oligomeric forms. By assuming a model of pseudo-equilibrium like monomers ↔ aggregates, NMR describes the conformational preferences of the monomeric entity, and in the case of fast exchange regime of the monomer with the NMR transparent large aggregates, the technique reads the conformational tendency of the monomer mixed to its memoire of the structure adopted into the aggregates [36]. Barrera and co-authors, in a very recent commentary, re-analyzed their dynamic simulation studies of P31–43 and found ‘remarkable agreement’ between experimental NMR structures and those visited by the monomer during the simulations as well as those adopted in the simulated oligomeric forms, thus, suggesting “that P31–43 suffers very minor conformational changes when passing from monomeric to oligomeric states” [22].

This study deals with the structural comparison among P31–43 and the immunodominant peptides (Table 1). With the exception of DQ2α2 (PQPELPY), of which a strict analogue (PQPQLPY) was characterized in free form by solution NMR [37], all the others are structurally known only in complex with HLA-DQ. Because containing Pro residues, all those peptides are expected to exhibit in free form the same *cis*/*trans* isomerism and conformers distribution as found for P31–43 in solution. They are flexible enough to adapt to the binding pocket and at the same time rigid enough, thanks to the prolines, to minimize the negative entropic variation associated with the binding. When bound to HLA-DQ, they all adopt a PPII-like structure inside the binding pocket. That appears as an interesting property of the groove topology that, shared by different HLA-DQs, forces different gliadin epitopes into a strictly similar conformation. Indeed, the backbone superimpositions among the gliadin ligands show very low RMSD values ranging from 0.178 to 0.425 Å, if we consider for example the DQ2.5-glia-α vs a series of other DQ2.5 epitopes (Table S1). When the docking of P31–43 into HLA-DQ2.5 was simulated, it was found that the peptide, both in the experimental structure or modelled in PPII, engages with the groove causing a number of interactions sensibly lower than those established by the DQ2.5-glia-α1 [17]. P31–43 shows an arrangement of its prolines different from that of DQ2.5-glia-α1 and this plays against the binding with HLADQ2.5. P31–43 does not appear to be a good binder for HLA-DQ8 either. By sequence and amino acid composition, P31-43 is very different from the epitopes of DQ8 and therefore unsuitable to engage the interactions specified by HLA-DQ8 binding groove.

While the structural reasons why P31–43 is not an adaptive immune player via HLA-DQ molecules are apparently deciphered, those underlining the many mechanisms in which it is involved as innate immune agent remain to be clarified. Gomez-Castro and co-authors [14] propose that P31–43, due to its ability to self-aggregate, acts at oligomeric state in triggering the NPRP3 inflammosome and thus the intestinal pathology. Herrera and co-authors [23] suggest that the formation of P31–43 nanostructures induces proinflammatory effects and subsequent damage at the intestinal mucosa in CeD. We think that last decades of experimental and simulative works made clear that all peptides adopt a distribution of conformations in solution or fluid media, that in dependence of the concentration, they participate to apparent equilibria with ordered/un-ordered aggregates floating with them in the common media, and that, opportunely treated, all peptides can ‘solidify’ ordinately in the form best adhering to their nature, i.e., α or β-fibers.

Although the observation that the toxic behavior of P31–43 could be due to its self-aggregation ability represents an interesting model, we observe that knowing in which proportion the peptide is distributed among the various oligomeric forms depends upon the environments and this is an issue difficult to be estimated in cells. Indeed, we suggest that P31–43, together with almost all other protein fragments, represent objects that may act in several ways following several routes in reason of the cell district they enter, the concentration they are able to reach there, and the molecular or supramolecular entities they come into contact with.

In conclusion, it is interesting to note that starting from gliadin proteins, known to be very monotonous macromolecules, made up from blocks of similar sequences repeated many times, the enzymatic digestion releases in gut fragments that provoke different biological responses in CeD individuals [16]. P31–43, a not adaptive immune player, shares a similar content in proline residues, similar adaptability to polyproline II structure, but a different positioning of the proline residues along the sequence with the adaptive ones. This last issue disfavors a productive binding to HLA-DQ2.5 and thus P31–43 access to the adaptive immune route.

## 4. Materials and Methods 

### 4.1. NMR Analysis

P31–43 peptide was purchased from Inbios (Naples, Italy). Deuterated solvents, such as D2O, (99,8% isotopic purity) and sodium dodecyl-d_25_ sulphate (SDS-d25, > 98 atom % D) were purchased from Sigma-Aldrich (Milan, Italy). NMR measurements of P31–43 in aqueous solution were obtained at 600MHz proton resonance frequency as previously reported [17]. Briefly, NMR characterizations of P31–43 were performed at 298 K in a H_2_O/D_2_O 90:10 (*v*/*v*) mixture and in sodium dodecyl sulfate 150 mM (SDS) at pH 4.6 ± 0.1 where the peptide has a net electrical charge equal to zero. Proton resonance assignments were obtained by analyzing the suite of bi-dimensional DQFCOSY, TOCSY, NOESY and ROESY experiments. Proton assignments of the amino acid spin systems and 1D proton spectrum of P31–43 in aqueous as well as in SDS media are reported [17]. The percentage of P31–43 in ‘all *trans*’ structure was estimated at about 60–65% of the total population in water solution. The three-dimensional models were obtained by following the classical protocol consisting of: Assignment of the proton chemical shifts, integration of the dipolar effects (NOE), conversion of NOE intensities into inter-proton distances, and calculation of peptide structures compatible with the entire set of distance restraints by using CYANA software [38]. In order to characterize peptide flexibility and weight of different conformations, CYANA structures were clustered by similarity using UCSF Chimera program (version 1.14) [24].

### 4.2. Structure Comparison of Gluten Peptides

All the X-ray structures of binary HLA-DQ/gluten peptides and ternary HLA-DQ/gluten peptides/T-cell receptor complexes published on the PDB databank on October 2020 (Table 1) were downloaded and analyzed. Particularly, all the gluten fragments inside HLA-DQ grooves were extracted and structurally compared each other. First comparisons consider the differences among the structures of the same sequence in the same HLA-DQX, then of the same sequence in different HLA-DQ. Then, the structural differences between different gliadin ligands were analyzed. Finally, structural differences between those and related bacteria peptide mimetic were tested. All structure comparisons were obtained by peptide superimpositions based on a principle of register correspondence by using PyMOL software (http://www.PyMOL.org) and the open source software MOLMOL 2K.2.0 [39].

## Figures and Tables

**Table 1:** The list of Protein Data Bank (PDB) structures of binary and ternary complexes involving human leucocyte antigen (HLA)DQ, gluten peptides and T-cell receptor (TCR) updated to October 2020.

PDB ID	HLA-II	Binder	Complex	References
6U3M	DQ2.5	mimic glia- α1a	binary	[30]
6U3N	DQ2.5	mimic glia- α1a	ternary (TCR LS2.8/3.15)	[30]
6U3O	DQ2.5	mimic glia- α2a	ternary (TCR JR5.1)	[30]
6PY2	DQ2.2	glut-L1	ternary (TCR T594)	[31]
6MFF	DQ2.5	glia-ω1	binary	[5]
6MFG	DQ2.5	glia-α1a	binary	[5]
4OZI	DQ2.5	glia-α1a	ternary (TCR S2)	[1]
1S9V	DQ2	α1	binary	[9]
4OZF	DQ2	α2	ternary (TCR JR51)	[1]
4OZG	DQ2	α2	ternary (TCR d2)	[1]
4OZH	DQ2	α2	ternary (TCR s16)	[1]
5KS9	DQ8	glia-α1	ternary (Bel502 TCR)	[32]
5KSA	DQ8.5	glia-γ1	ternary (Bel602 TCR)	[32]
5KSB	DQ8.5	glia-γ1	ternary (T15 TCR)	[32]
4Z7U	DQ8	glia-α1	ternary (S13 TCR)	[33]
4Z7V	DQ8	glia-α1	ternary (L3−12 TCR)	[33]
4Z7W	DQ8	glia-α1	ternary (T316 TCR)	[33]
4GG6	DQ8	glia-α1	ternary (SP3.4 TCR)	[7]
2NNA	DQ8	glia-α1	binary	[34]
4D8P	DQ2.3	peptide	binary	[35]

## Supplementary Materials

Supplementary materials can be found at https://www.mdpi.com/1422-0067/21/23/9301/s1.

## Abbreviations

CeD	Celiac disease
HLA	Human leucocyte antigen
TCR	T-cells antigen receptor
TG	Transglutaminase
PPII	Polyproline II
