# Supplementary material for: Structural Perspective of Gliadin Peptides Active in Celiac Disease
Source: Int J Mol Sci. 2020 Dec 6;21(23):9301. doi: 10.3390/ijms21239301 (PMC7731278; doi:10.3390/ijms21239301)
Supplement: Supplementary file 1 [file ijms-21-09301-s001.pdf]

Supplementary Materials

Structural perspective of gliadin peptides active in Celiac disease

Lucia Falcigno[1,*], Luisa Calvanese[1], Mariangela Conte[2], Merlin Nanayakkara[2],

Maria Vittoria Barone[2] and Gabriella D'Auria[1,*]

[1] University of Naples Federico II, Department of Pharmacy, via Mezzocannone 16, 80134 Naples, Italy.
[2] University of Naples Federico II, Department of Translational Medical Science (section of Pediatrics) and ELFID (European Laboratory for the Investigation of Food Induced Diseases), 80131 Naples, Italy.
* Correspondence: (G.D.) gabriella.dauria@unina.it, (L. F.) falcigno@unina.it

Table S1 (A-F)

Quantitative measure of the similarity, i.e. Root Mean Square Deviation (RMSD, Å) between superimposed atomic coordinates of peptides. Superimpositions obtained by using PyMOL software (http://www.PyMOL.org). The row and column headers refer to the PDB codes of the gliadin peptides bound to HLA-DQ in binary or ternary complexes. P3143-PPII refers to P31-43 peptide modelled in polyproline II (PPII).

Table S1A. DQ2.5-glia-α1 vs. other gluten peptides. Sequence based alignment (RMSD Å)

	**6mfg_pepF**	**4ozi-pepI**	**6mmf_pepC**	**6u3m-pepE**	**P3143-PPII**
**1s9v-pepC**	0.265	0.562	0.299	0.403	2.441
**6mfg_pepF**		0.345	0.219	0.280	2.484 (ω1)
**4ozi-pepI**			0.404	0.385	2.560
**6mmf_pepC**				0.230	2.438
**6u3m-pepE**					2.454

Table S1B. DQ2.5-glia-α1 vs. other gluten peptides. Structure based alignment (RMSD Å)

	**6mfg_pepF**	**4ozi-pepI**	**6mmf_pepC**	**6u3m-pepE**	**P3143- PPII**
**1s9v-pepC**	0.178	0.518	0.343	0.344	1.30
**6mfg_pepF**		0.337	0.289	0.172	1.14 (ω1)
**4ozi-pepI**			0.400	0.425	1.28
**6mmf_pepC**				0.290	1.01
**6u3m-pepE**					0.951

Table S1C. DQ2.5-glia-α2 vs. other gluten peptides. Sequence based alignment (RMSD Å)

	**4ozg-pepI**	**4ozh-pepJ**	**6u3o-pepI**	**P3143- PPII**
**4ozf-pepJ**	0.519	0.308	0.150	0.793
**4ozg-pepI**		0.279	0.165	0.834
**4ozh-pepJ**			0.240	0.753
**6u3o-pepI**				1.029

Table S1D. DQ2.5-glia-α2 vs. other gluten peptides. Structure based alignment (RMSD Å)

	**4ozg-pepI**	**4ozh-pepJ**	**6u3o-pepI**	**P3143-PPII**
**4ozf-pepJ**	0.610	0.369	2.150	1.314
**4ozg-pepI**		0.322	0.414	1.440
**4ozh-pepJ**			0.526	1.352
**6u3o-pepI**				2.148

Table S1E. DQ8-glia-α1 vs. other gluten peptides. Sequence based alignment (RMSD Å)

	5ks9-pep	4z7v-pepI	4z7u-pepI	4z7w-pepI	4gg6-pepI	5ksb-pepI	P3143-PPII
**2nna-pepC**	0.350	0.360	0.315	0.237	0.389	2.086	0.529
**5ks9-pepI**		0.356	0.338	0.416	0.346	0.981	0.417
**4z7v-pepI**			0.209	0.297	0.313	2.077	0.366
**4z7u-pepI**				0.315	0.297	0.978	0.368
**4z7w-pepI**					0.391	0.943	0.555
**4gg6-pepI**						2.050	0.372
**5ksb-pepI**							1.522 (γ1)

Table S1F. DQ8-glia-α1 vs. other gluten peptides. Structure based alignment (RMSD Å)

	5ks9-pep	4z7v-pepI	4z7u-pepI	4z7w-pepI	4gg6-pepI	5ksb-pepI	P3143-PPII
**2nna-pepC**	0.303	0.422	0.312	0.258	0.378	1.253	1.155
**5ks9-pepI**		0.274	0.320	0.436	0.445	0.501	1.792
**4z7v-pepI**			0.214	0.358	0.296	1.286	1.002
**4z7u-pepI**				0.380	0.348	1.439	1.355
**4z7w-pepI**					0.321	0.420	1.626
**4gg6-pepI**						1.207	0.856
**5ksb-pepI**							1.874 (γ1)
